# Morphometric characteristics of the knee are associated with the injury of the meniscus

**DOI:** 10.1186/s13018-022-03380-2

**Published:** 2022-11-19

**Authors:** Peixu Wang, Fuqiang Gao, Wei Sun, Zirong Li, Xinjie Wu, Lijun Shi, Xin Xu, Tengqi Li, Xiaoyu Fan, Chengxin Li, Zhizhuo Li

**Affiliations:** 1 Department of Orthopedics, China-Japan Friendship Hospital, China-Japan Friendship Institute of Clinical Medicine, Chinese Academy of Medical Sciences, Peking Union Medical College, Graduate School of Peking Union Medical College, Beijing, 100029 China; 2 Department of Orthopedics, Beijing Key Laboratory for Immune-Mediated Inflammatory Diseases, China-Japan Friendship Hospital, Peking Union Medical College, Beijing, 100029 China; 3 China-Japan Friendship Hospital, Peking University, Beijing, 100029 China; 4 Department of Orthopaedic Surgery, Perelman School of Medicine, University of Pennsylvania, Philadelphia, USA

**Keywords:** Meniscal injury, Anatomy, Knee, Meniscus

## Abstract

**Background:**

To assess the geometrical risk factors for meniscal injuries. We hypothesized that the narrowness of the intercondylar notch and the smaller tibial spine could increase the risk of meniscal injuries.

**Methods:**

We retrospectively studied two hundred and seven patients examined for knee magnetic resonance images. Two experienced orthopedists evaluated the severity of meniscal injuries. The notch width, bicondylar notch width, notch width index, condyle width of the femur, tibial spine height, and intercondylar angle were measured in magnetic resonance image slides by two blinded orthopedists.

**Results:**

A total of 112 patients with a meniscus injury and 95 patients were as healthy control in all two hundred and seven patients. The NWI (*P* = 0.027) in patients with meniscus injuries was significantly different from the control group. A 1 SD (0.04 mm) increase in NWI was associated with a 0.4-fold increase in the risk of meniscal injury. A 1 SD (0.04 mm) increase in NWI was associated with a 0.64-fold increase in the risk of grade 3 meniscal injury. Furthermore, NWI and medial spine height are decreased significantly in grade 2 (*P* < 0.05) meniscal injury than in other grades. The medial spine height was significantly decreased in the meniscal injury group (*P* = 0.025), and the decrease in medial spine height would increase the risk of meniscal injury (OR = 0.77) and grade 3 meniscal injury (OR = 0.8).

**Conclusions:**

The stenosis of the femoral intercondylar notch and small medial tibial spine is risk factors of meniscal injury. The decreased NWI and the medial tibial spine height were also associated with the severity of the meniscal injury.

**Supplementary Information:**

The online version contains supplementary material available at 10.1186/s13018-022-03380-2.

## Background

The injury of the meniscus is one of the most common orthopedic issues worldwide. It is commonly seen in athletes with pivoting maneuvers. Sometimes patients performing low-impact sports can also be injured [1].

The meniscus and the anterior cruciate ligament (ACL) are closely intertwined. The medial meniscus is a secondary stabilizer to ACL in ACL-deficient knees [2–10]. Patients with ACL deficiency have an abnormality of anterior tibial translation, increasing the incidence of meniscal injuries [4–10].

Geometric characteristics of the knee were widely studied in patients with ACL injuries [11–16]. Previous studies have shown the relative parameters can predict the risk of ACL injuries [14–18]. It was found that the stenosis of the femoral notch was significantly associated with ACL injuries [14–16, 19, 20]. Several parameters are related to the width of the femoral notch [11, 12], such as the notch width (NW), bicondylar notch width (BCW), the notch width index (NWI), condyle width of the femur, tibial spine height, and intercondylar angle. However, limited prior studies evaluated the anatomical risk factors for the injury of the meniscus [21–24].

This study is set out to investigate the relationship between the anatomical characteristics of the knee and the meniscal injury using magnetic resonance imaging (MRI) data. We hypothesized that the geometrical features of the knee such as NW, BCW, NWI, condyle width, spine height, and intercondylar angle are associated with the injury of the meniscus.

## Methods

### Study participants

This study was a retrospective case–control study including 207 patients with or without meniscal injury from January 2015 to January 2020. All patients were diagnosed by MR images and confirmed by two experienced orthopaedists. Patients without any sign of meniscal injury were included as the control group. The excluding criteria were as follows: patients with femoral or tibial fractures, previous surgery of knee or ligaments, deformity of the knee. All participants recorded age, sex, and with or without ACL injury or rupture. The ethics committee approved this study of China–Japan Friendship Hospital.

### Measurement of magnetic resonance image

For each participation included, the 1.5 Tesla Knee MRIs were collected. The thickness of the slides was 4 mm. DICOM MRI images were viewed and measured using OsiriX Software (Pixmeo, Geneva, Switzerland, version 12.0.2).

Two experienced orthopaedists examined meniscal injuries, and the high signal intensities of the meniscus were classified using the three-grade staging system introduced by Fishcher et al. [25]. Grade 1: A small focal area limited the high-intensity signal on T2-weighted images. Grade 2: the high-intensity signal presented as a linear area but did not extend to the articular surface. Grade 3: the abnormal signal extended to at least one articular surface, indicating meniscal injury.

**Fig. 1:**
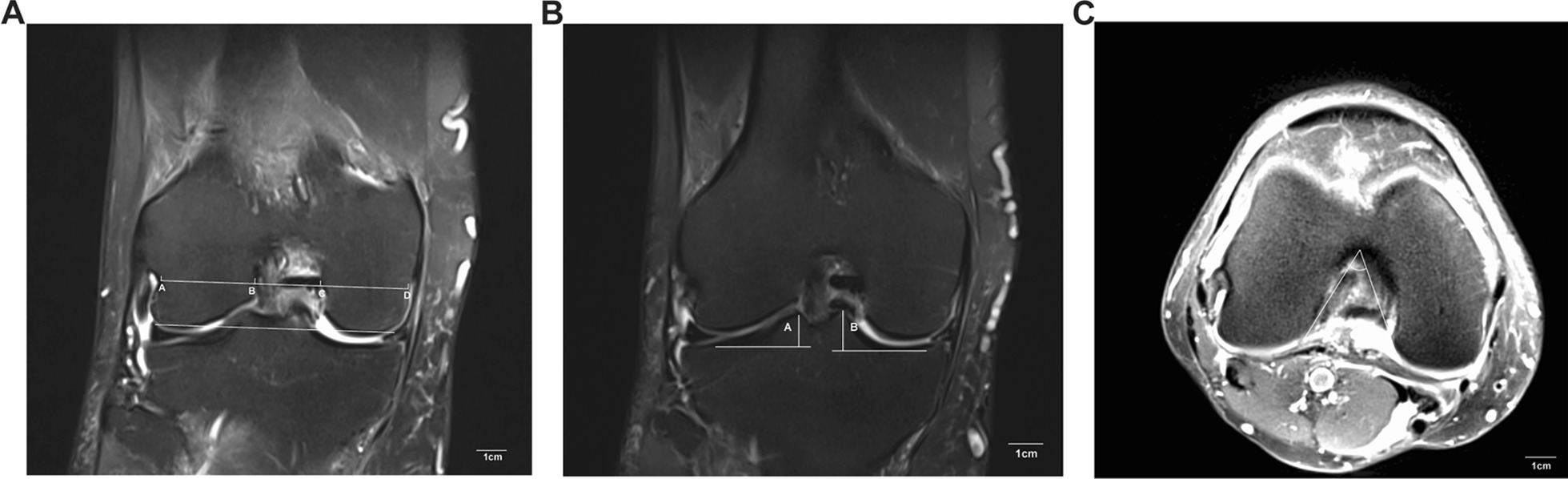
**A** The measurement of NW, BCW, and condyle width on T2 Coronal view of MRI. A line was drawn through the most inferior boarder of the femoral condyles, and a parallel line was sketched at the level of the popliteal groove. The distance between AB and CD indicated the lateral and medial femoral condyle width, respectively. The distance between AD denoted as the BCW. The distance between BC represented as the NW. **B** The measurement of the height of medial spine on T2 Coronal view of MRI. The height was defined as the distance from the peak of the tibial spine to the point of the most concave in the tibia. **C** The intercondylar angle was measured in proton density-weighted axial magnetic resonance images. The angle was shaped by two lines which are from the top of the intercondylar notch to the most inferior border of the intercondylar notch at both medial and lateral condyles

The NW and BCW were measured using the method introduced by Domzalski et al. [26]. The BCW was measured at the level of the popliteal grove in a T2-weighted coronal magnetic resonance image. The line was drawled from the lateral condyle of the femur, parallel to the joint line and connected to the distal femoral condyles. The NW was measured from the most inferior margins of the borders of the intercondylar notch. Both the width of the medial and lateral femoral condyle were measured at the same level (Fig. 1A). The NWI represented the ratio of NW to the BCW. The height of the tibial spines was measured on the T2-weighted mid-coronal magnetic resonance image, defined as the single slice that presented the largest area of the tibial spine. The greatest width of the tibial was chosen if the size of the spine in the two slices was similar (Fig. 1B). The intercondylar angle was shaped by two lines from the top of the intercondylar notch to the most inferior border of the intercondylar notch at both medial and lateral condyles in proton density-weighted axial magnetic resonance images (Fig. 1C).

All the measurements were made twice by two experienced orthopedists. The average of each measurement was calculated.

### Statistical analysis

For continuous variables, the normal distribution test was performed using the Kolmogorov–Smirnov test. The unpaired *t*-test was used if the variable were to compare the difference between the group of meniscal tears and the normal group. The one-way analysis of variance (ANOVA) with post hoc test (LSD) was used to explore the underlying relationship between the grade of the meniscal tear and the age, NW, BCW, NWI, the height of medial spine peak, condyle width, and intercondylar angle which was presented as mean ± standard deviation. For categorical data, Chi-square tests were used to analyze the relationship of meniscal injuries and the sex, the number of patients with injury or rupture of ACL, which presented as amount and proportions. To analyze the most highly associated factors associated with the meniscal injury and the grade of meniscal injury, binary and ordinal logistic regression with the Enter method were used. Correlated independents were excluded to avoid the influence of these independents on the risk of meniscal injury or the grade of meniscal injury. The variable included in the logistic regression were either associated with the risk of meniscal injury or the grade of meniscal injury independently or had clinical associations with the dependent. All data were analyzed using SPSS software, version 27.0 (SPSS Inc., Chicago, IL). The significance level for all analyses was set at *P* < 0.05.

The interclass correlation coefficient (ICC) was calculated to evaluate the intra- and interobserver reliability. The ICC value greater than 0.9 was considered excellent, and a good value was defined between the 0.8 and 0.9 [27].

## Results

### Patients characteristics

In total, 207 patients were included in this study, 112 patients with the injured meniscus, and 95 patients as the control group. Among them, 119 were female, and 88 were male, with a mean age of 48.63 ± 15.53. Among the patients being studied, 59 (32.37%) with an ACL injury, 45 (21.74%) with ACL rupture were observed in 74 (35.75%) patients (Table 1). The reproducibility of all the measurements in magnetic resonance images was excellent, with an average ICC of 0.96 (from 0.91 to 0.99). Details are shown in Additional file 1: Table S1.

**Table 1:** Patient characteristics and the difference between the meniscal injury and control groups

Variable	Total (*n* = 207)	Control group (*n* = 95)	Meniscal injury group (*n* = 112)	*P* value
Gender				
Male	88 (63.63%)	38 (40.00%)	50 (44.64%)	0.501
Female	119 (57.49%)	57 (60.00%)	62 (55.35%)	
Age, years	48.63 ± 15.53	43.18 ± 14.96	53.26 ± 14.53	< 0.001
ACL injury	59 (32.37%)	26 (27.37%)	33 (29.46%)	0.811
ACL rupture	45 (21.74%)	18 (18.95%)	27 (24.11%)	0.370
NW, mm	18.7 ± 2.43	19.02 ± 2.31	18.43 ± 2.5	0.081
BCW, mm	68.38 ± 6.33	68.11 ± 7.22	68.61 ± 5.5	0.574
NWI	0.28 ± 0.04	0.28 ± 0.05	0.27 ± 0.04	0.027
Medial condyle width, mm	23.29 ± 2.56	23.01 ± 2.59	23.53 ± 2.52	0.145
Lateral condyle width, mm	41.19 ± 208.17	27.27 ± 5.62	53 ± 283	0.199
Medial spine height, mm	9.74 ± 1.42	9.98 ± 1.35	9.54 ± 1.45	0.025
Lateral spine height, mm	7.58 ± 1.21	7.59 ± 1.22	7.58 ± 1.2	0.939
Intercondylar angle, °	35.93 ± 6.22	35.57 ± 7	36.25 ± 5.49	0.435

*ACL* anterior cruciate ligament, *NW* femoral notch width, *BCW* bicondylar width, *NWI* notch width index

### Correlation between the meniscus tear and knee morphometrics

Compared with the control group, the NWI (*P* = 0.027) in meniscus injuries patients was significantly different from that in patients without it. In contrast, there was no significant difference between the two groups regarding NW (*P* = 0.081), BCW (*P* = 0.574), both medial and lateral condyle width (*P* = 0.145, *P* = 0.199), and lateral spine height (*P* = 0.925). Furthermore, meniscal injury's medial spine height (*P* = 0.025) was significantly different from the control group (Table 1). In the binary logistic regression modeling adjust for gender, age, NWI, medial condyle width, lateral condyle width, medial spine height, lateral spine height, and intercondylar angle (Table 2), five variables were found to be associated with increased odds of meniscal injury: gender (OR 3.43, 95% CI 1.23–9.55), age (OR 1.06, 95% CI 1.04–1.09), NWI (OR 0.4, 95% CI 0.2–0.78), medial spine height (OR 0.77, 95% CI 0.61–0.98) and intercondylar angle (OR 1.05, 95% CI 1–1.11). A 1 SD (0.04 mm) increase in NWI was associated with reduced risk of meniscal injury (OR 0.4, 95% CI 0.2–0.78).

**Table 2:** Associations between knee morphometrics and the risk of meniscal injury

Variable	OR	95% CI	*P* value
Gender (male)	3.43	1.23–9.55	0.018
Age, years	1.06	1.04–1.09	< 0.001
NWI (per SD)	0.4	0.2–0.78	0.007
Medial condyle width, mm	1.1	0.91–1.32	0.331
Lateral condyle width, mm	0.87	0.76–1.01	0.058
Medial spine height, mm	0.77	0.61–0.98	0.032
Lateral spine height, mm	1.01	0.76–1.33	0.964
Intercondylar angle, °	1.05	1–1.11	0.066

*ACL* anterior cruciate ligament, *NWI* notch width index, *OR* odds ratio

### Correlation between the grade of meniscus injury and knee morphometrics

The pairwise comparison was performed to identify the difference between the grades of meniscal injury and the control group. Significant differences were observed in age, ACL rupture, NWI, medial condyle width, and medial spine height (Table 3). All three grades showed significant differences compared with the control group regarding the age of patients, respectively. The number of patients with grade 3 meniscal injury and ACL rupture was significantly different from grade 1. The NWI in patients with grade 2 meniscal injury was significantly lower than that of the control group. No significance was found among the 3°.

**Table 3:** The difference between three grades of meniscal injury and the control group

Variable	Control (*n* = 96)	Grade 1 (*n* = 19)	Grade 2 (*n* = 50)	Grade 3 (*n* = 42)
Gender (male)	38 (39.60%)	10 (52.60%)	16 (32.00%)	24 (57.10%)
Age, years	43.3 ± 14.93	51.63 ± 11.95_a_	55.38 ± 13.06_b_	51.43 ± 17.17_c_
ACL injury	27 (28.1%)	2 (10.5%)	10 (20%)	16 (38.1%)
ACL rupture	19 (19.8%)	1 (5.3%)	8 (16%)	17 (40.5%)_e_
NW, mm	19.05 ± 2.32	18.7 ± 2.7	18.31 ± 2.52	18.37 ± 2.39
BCW, mm	68.11 ± 7.18	68.93 ± 6.56	68.23 ± 5.03	68.92 ± 5.69
NWI	0.28 ± 0.05	0.27 ± 0.04	0.27 ± 0.03_b_	0.27 ± 0.04
Medial condyle width, mm	22.97 ± 2.6	23.99 ± 2.6	22.88 ± 2.4	24.2 ± 2.39_cf_
Lateral condyle width, mm	27.27 ± 5.59	26.22 ± 3.68	26.3 ± 2.28	26.79 ± 3.48
Medial spine height, mm	9.98 ± 1.34	9.98 ± 1.6	9.14 ± 1.23_bdf_	9.81 ± 1.54
Lateral spine height, mm	7.59 ± 1.21	7.71 ± 1.15	7.5 ± 1.25	7.63 ± 1.2

*ACL* anterior cruciate ligament, *NW* femoral notch width, *BCW* bicondylar width, *NWI* notch width index

Furthermore, we found significant differences in the medial condyle width between grade 3 and the control group and grade 3 and grade 2. In addition, medial spin height also showed a significant difference between grade 2 and the control group, grade 2 and grade 1, grade 2 and grade 3, respectively. In the ordinal logistic regression modeling adjust for gender, age, NWI, medial condyle width, lateral condyle width, medial spine height, lateral spine height, and intercondylar angle (Table 4), five variables were found to be associated with an increased degree of meniscal injury: gender (OR 0.29, 95% CI 0.12–0.70), age (OR 1.05, 95% CI 0.2–1.05), NWI (OR 0.64, 95% CI 0.46–0.89), lateral condyle width (OR 0.88, 95% CI 0.78–0.99) and medial spine height (OR 0.8, 95% CI 0.66–0.99). A 1 SD (0.04 mm) increase in NWI was associated with reduced risk of greater grade meniscal injury (OR 0.64, 95% CI 0.46–0.89). Similar to the results from risk factors of meniscus tear, the decrease in age, NWI, lateral condyle width, and medial spine height could be associated with a higher degree of meniscal injury since the ORs of these factors were less than 1.

**Table 4:** Associations between knee morphometrics and the degree of meniscal injury

Variable	OR	95% CI	*P* value
Gender (male)	0.29	0.12–0.70	0.006
Age, years	1.05	1.03–1.07	< 0.001
NWI (per SD)	0.64	0.46–0.89	0.008
Medial condyle width	1.11	0.94–1.3	0.216
Lateral condyle width	0.88	0.78–0.99	0.061
Medial spine height	0.8	0.66–0.99	0.037
Lateral spine height	0.99	0.78–1.25	0.936
Intercondylar angle	1.05	1–1.1	0.055

*NWI* notch width index, *OR* odds ratio, *CI* confidence interval

## Discussion

The major discovery of our study was the correlation of the geometrical parameters of the knee and the meniscal injuries. Specifically, the decrease in NWI and medial spine height was significantly correlated with the increased risk of meniscal injury compared with the control group. Moreover, NWI and medial spine height are also significantly related to the severity of the meniscal injury. Although statistical significance was not found in grade 1 and grade 3 compared with the control group, the result showed lower NWI in these two groups. It is worth noting that although NWI is statistically significant lower compared to the control group, the clinical significance may not differ so much since the difference of mean value between two groups was small. This small difference can also be created by the measurement in MRI. Furthermore, we also confirmed the meniscal injury was correlated with sex and age. Ordinal regression also verified the relationship between genders, and age, which is related to the severity of the meniscal injury. Contrary to our proposition, NW, BCW, medial and lateral condyle width, and lateral spine height are not associated with the risk of meniscal injury. Although these are important parameters of the knee morphology, out results show that they are not relevant with respect to the meniscal injury.

The findings of our study reveal that meniscal injury and its grade were associated with NW and NWI, which are similar to the conclusions of previous results on ACL injuries [12, 14]. In 1938, Palmer first recognized the narrowness of intercondylar was associated with the ACL injury [28]. Souryal et al. portrayed a method of measuring the intercondylar width, the NWI on the plain radiograph [13] and found the correlation between the stenosis of the intercondylar notch and the injury of ACL. Previously studies have concluded that the femoral NW and NWI, as two-dimensional parameters, can effectively evaluate the femoral notch's size [29]. Previous studies demonstrated that the stenosis of intercondylar notch increases the risk of ACL injury [12, 14]. A narrow space of intercondylar notch tends to house a relatively small volume ACL. The strength of the ligament was decreased and consequently led to the predisposition of ACL injury [19, 20]. As the knee is over-bent or rotated, a narrow intercondylar notch leads to an impact between the lateral wall of the femoral intercondylar and ACL. This phenomenon was more evident when the knee was externally rotated or at the position of flexion-valgus. As the impact of ACL harms the fiber bundles, the intensity of ACL decreases and prone to be injured [19, 20]. On the other hand, the correlation between the NW or NWI and the risk of ACL injury was controversial [12, 30]. Some studies showed no significant relationship between the narrow intercondylar notch and the risk of ACL injury [16, 31]. A meta-analysis conducted by Li et al. showed the NW was significantly narrower in ACL injury cases, and the result was consistent with different ethnicity and sex [32]. However, the NW varies a lot among patients, and the method used for measurement can generate discrepancies. NWI can better represent the volume of the intercondylar notch by excluding the difference in height, weight, individuals, and measurements. However, as age increases, the NWI decreases, as Domzalski et al. reported [26]. With different ethics and measurements, the cut-off value for NWI reported in previous studies was different from 0.18 to 0.20 [13, 14, 26].

The tibial spine is located at the center of the tibial femoral articular surface through weight-bearing activity. The medial tibial spun has the highest contact pressure in a load-bearing knee. Previous studies described the anatomical relationship between the ACL and the tibial spine [33, 34]. Oka et al. [33] found the anterior part of the medial tibial spine was attached by the medial margin of the ACL. A similar result was reported by Tensho et al. [34] using 3D-CT. They concluded that some ACL fibers were connected with the medial tibial spine, whereas no similar connection was found in the lateral tibial spine. McDonald et al. [35] observed significantly increased intersegmental load conveyed between the medial femoral condyle and the ipsilateral tibial spine. Levins et al. [36] also found the morphology of both medial and lateral tibial spine can influence ACL injury. The decrease in the height of tibial spines leads to the increase in anterior translation and the internal rotation of the tibia, subsequently increasing the strain of ACL and finally leading to the injury of the ACL [37]. A similar conclusion was also found by Sturnick et al. [38], and the decreased medial spine on height males increased the risk of ACL injury. In contrast, a significant correlation was not observed on females. Similar to the results from studies in ACL injuries, we found the decrease in medial tibial spine height can increase the risk of meniscal injuries. And in patients with grade 2 meniscal injury, the height of the medial tibial spine is significantly decreased compared to another degree of injury and the control group.

The intercondylar angle was another parameter that described the intercondylar notch. However, the investigation of intercondylar angle was poor in ACL-related papers. Alentorn et al. [17] found the decrease in intercondylar angle would increase the risk of ACL injuries, and they suggested a 50° of cut-off value. The same cut-off value was accepted by Stein et al. [18], but they found no association between the angle and the risk of ACL injuries. Alentorn et al. found the intercondylar notch angle was significantly narrower in ACL injury patients. Therefore, they considered the intercondylar angle a more useful parameter for describing the narrow intercondylar notch [39]. A similar result was concluded by Raja et al. [40]. In this study, we found intercondylar angle did not associate with meniscal injury.

Females are more apt to have ACL injuries than males, and the anatomical structures were different in females [41]. The results of Wolters et al. [42] showed a narrower intercondylar notch in women, whereas results from Eck et al. [43] concluded that there were no differences in NWI between genders. In addition, the risk of ACL injury increased with age, as the results from Snoeker et al. [44]. The difference in age and gender can also be found in the meniscus injury. The prevalence of meniscal injury increased with the age of patients [45]. On the other hand, the incidence of acute meniscal injury decreased with age [46]. The difference between gender in patients with meniscal injury remains controversial. In athletes, meniscal injury was more easily found in males than females [47]. In contrast, female athletes have a higher risk for medial meniscus posterior root tears [48]. Our study found that the number of female patients is more prominent than male, although no significant difference was found between the groups and the severity of the injury. However, the opposite result was found after being analyzed by different severity of the injury. It may occur due to the relatively small amount of patients in each grade of injury and lead to potential bias.

The ACL and meniscus have inseparable correlations when it comes to the injury of the knee [2–6, 12]. Meniscus tears have been reported in 40–82% of ACL tears, and the medial meniscus was more likely to be injured compared with the lateral meniscus [45]. Previous studies considered the medial meniscus also has a restraining effect on the anterior tibial translation ACL. Shybut et al. [4] found the tibial translation change increased significantly with ACL-deficiency knee, which can lead to the injury of the meniscus. With the deficient ACL, patients were prone to have more significant internal rotation, and the meniscal translation increased compared to in the intact state [5]. Levy et al. showed that compared to the lateral meniscus, the medial meniscus has a significant posterior wedge effect and is firmly connected to the tibial plateau with capsular attachments [2]. The anterior tibial translation insignificantly increased after performing lateral meniscectomy in an ACL-deficient knee [3]. Similar to the results of Levy et al., multiple prior studies have concluded the medial meniscus was a secondary stabilizer to ACL at the process of anterior translation [8, 9, 12]. After studying cadavers with both lateral and medial meniscectomies, Musahl et al. [49] found medial meniscus played a more critical role in restraining the anterior tibial translation but had no effect on pivot shift. In comparison, the lateral meniscus exerted its influence on preventing rotational disability and cannot inhibit the anterior tibial translation. Arner et al. [5] found that with the decreased strength of ACL, the lateral meniscus had more mobility and was more likely to injure. The lateral meniscus tears often presented at the acute stage of ACL injury, whereas the medial meniscus tears were more likely to develop at the chronic stage. The greater translation can explain this in the lateral meniscus and the greater stress conducted to the medial meniscus [6]. On the other hand, the meniscus injury can also harm the stability of the ACL-deficiency knee. Shybut et al. [4] found the meniscal posterior root tears can further decrease the strength of the knee with ACL deficiency. The underlying mechanism was increased pivot-shift instability in those with injury of the lateral meniscus. The position of the meniscus could be altered after ACL reconstruction, which was reported in multiple studies [50, 51], and the ACL reconstruction can also restore the abnormal biomechanics such as meniscal shift [9]. These findings indicate the injury of the meniscus and the pathological extrusion was closely associated with ACL and can be influenced by the abnormality of the ACL.

Our studies have several limitations. First, our study measured the notch parameters and the tibial spine on segments of MRI, which only represent the intercondylar notch dimension at one slice. It cannot fully embody the overall volume of the intercondylar notch. Although a previous study found the two-dimensional measurement can effectively evaluate the volume of intercondylar notch [29], this measurement can lead to potential bias. However, due to the limitation of technology, the application of a three-dimensional measurement is restricted. Besides, our study was a retrospective case–control study, and the imaging data were acquired after the injury. The cause and consequences cannot be elucidated. For example, in those ACL-injured patients accompanied with meniscal injury, whether the narrowed intercondylar notch led to ACL injury first, therefore, led to meniscal injury or the narrowed intercondylar notch influenced the ACL and meniscus separately and directly remained unknown. Moreover, due to the characteristic of our study, the number of female and male participants was unequal. The gender difference can lead to different results in the prevalence of ACL injuries and meniscal injuries as previously reported [41, 44, 45]. Restricted by the limited time and resources, our study did not include the height and weight of subjects. Consequently, the BMI cannot be calculated. As previously reported, BMI is a risk factor of meniscal injury [44], and it may have a potential influence on our results. Future studies should take it into consideration. Only Chinese subjects were included in this study. The potential influence of ethnicity cannot be analyzed and should be considered in future studies.

## Conclusion

Our study shows that the stenosis of the femoral intercondylar notch and small medial tibial spine has an association with the increased risk of meniscal injury. The decreased NWI and the decreased medial tibial spine height were associated with the severity of the meniscal injury.

## Supplementary Information

**Additional file 1: Table S1.** Intra- and inter-observer reliability of measurements.

## Data Availability

All data generated or analyzed during this study are included in this published article and its Additional file 1.

## Abbreviations

ACL: Anterior cruciate ligament
NW: The femoral notch width
BCW: The bicondylar width
NWI: The notch width index
