# Supplementary material for: Morphometric characteristics of the knee are associated with the injury of the meniscus
Source: J Orthop Surg Res. 2022 Nov 19;17:498. doi: 10.1186/s13018-022-03380-2 (PMC9675146; doi:10.1186/s13018-022-03380-2)
Supplement: Supplementary file 1 — Additional file 1: Table S1. Intra- and inter-observer reliability of measurements. [file 13018_2022_3380_MOESM1_ESM.docx]

**Additional file 1: Table S1** Intra- and inter-observer reliability of measurements.

| **Variable** | **ICC** | **95% CI** |
| --- | --- | --- |
| NW | 0.97 | 0.04-1 |
| BCW | 0.99 | 0.56-1 |
| NWI | 0.99 | 0.26-1 |
| Medial condyle width | 0.91 | 0.01-0.98 |
| Lateral condyle width | 0.92 | 0.01-0.99 |
| Medial spine height | 0.97 | 0.03-1 |
| Lateral spine height | 0.98 | 0.05-1 |
| Intercondylar angle | 0.98 | 0.08-1 |
